# Squamous Cell Lung Cancer Associated With Systemic Sclerosis

**DOI:** 10.14740/jocmr2313w

**Published:** 2015-09-25

**Authors:** Nobuhiro Kanaji, Masaya Okuda, Hiroaki Dobashi, Tomohiro Kameda, Akira Tadokoro, Risa Wakiya, Norimitsu Kadowaki, Shuji Bandoh

**Affiliations:** a Department of Internal Medicine, Division of Hematology, Rheumatology and Respiratory Medicine, Faculty of Medicine, Kagawa University, Kagawa, Japan; b Department of General Thoracic, Breast and Endocrinogical Surgery, Faculty of Medicine, Kagawa University, Kagawa, Japan

**Keywords:** Squamous cell, Lung cancer, Systemic sclerosis, Interstitial lung disease, Pulmonary fibrosis, Interstitial pneumonia

## Abstract

We here describe a 50-year-old woman diagnosed with squamous cell lung cancer (SqLC) with underlying interstitial lung disease (ILD) 14 years after a diagnosis of systemic sclerosis (SSc). We reviewed the literature and collected 21 well-documented cases with SqLC associated with SSc including the present case. Several characteristics of SqLC associated with SSc have been found. First, the average age at diagnosis of SqLC is 57 years, which is much younger than that reported for patients without SSc. Second, SqLC could occur even in never or light smokers, although SqLC usually has a strong association with smoking history. Third, two-thirds of the available cases have ILD. In addition, SqLC developed in the area of ILD in most cases with ILD. Fourth, SqLC generally occurs after a long period from the diagnosis of SSc; the average of this interval reaches 12 years. It would be helpful to know these features so that physicians follow up and treat SSc patients adequately.

## Introduction

A recent meta-analysis has shown a higher incidence of cancer in patients with systemic sclerosis (SSc) (relative risk (RR) 1.75), and particularly a strong association with lung cancer has been estimated (RR 4.35) [1]. The most common histological type of lung cancer among patients with SSc is adenocarcinoma [2]. The occurrence of other histological types, including small cell and squamous cell lung cancer (SqLC), in patients with SSc appears to be rare [3].

On the other hand, SqLC has been reported to be the most common type of lung cancer associated with idiopathic pulmonary fibrosis (IPF), occupying a frequency of approximately 40% [4, 5]. In addition, after irradiation for breast cancer, the risk of developing lung cancer, particularly SqLC, increases in the ipsilateral lung field of irradiation [6]. These reports suggest that interstitial lung disease (ILD) may cause the development of SqLC.

It is well known that ILD is the most common finding in the lungs of patients with SSc [7]. In addition, it has been reported that patients with small cell lung cancer associated with SSc generally develop underlying ILD [3]. However, no reports have focused on SqLC associated with SSc. This report presents a well-documented case of SSc resulting in ILD and eventually in the development of SqLC. In addition, we review the literature to clarify the clinical features of SqLC associated with SSc.

## Case Report

**Figure 1:**
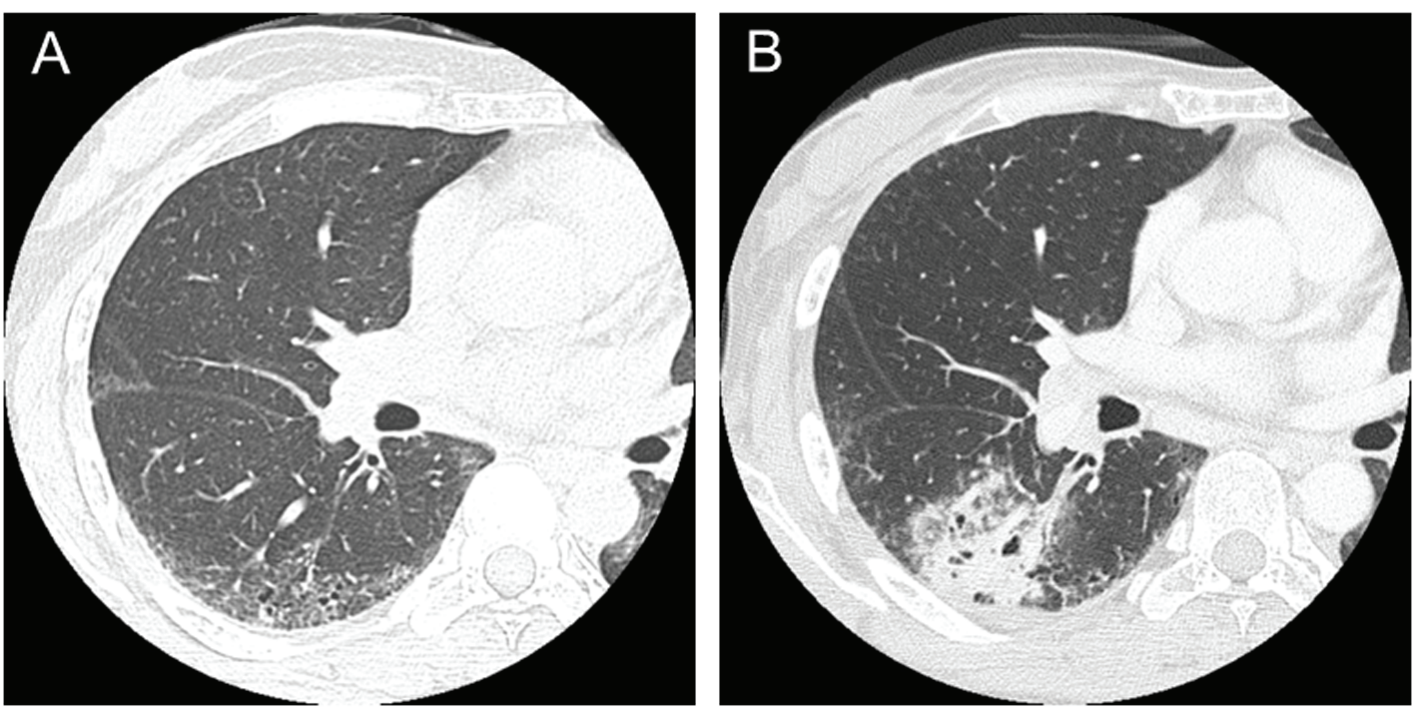
CT scans in a patient with systemic sclerosis. (A) Interstitial lung disease was observed in lower lobes in a 47-year-old patient. (B) Squamous cell lung cancer occurred in the area of interstitial lung disease in a 50-year-old patient.

A 36-year-old woman presented Raynaud’s phenomenon and sclerodactyly progressing for 1 year. Serological findings revealed positivity for anti-nuclear antibody (1:1,280, nucleus pattern) and anti-scl-70 antibody. No other specific antibodies were detected. She was diagnosed with SSc and started treatment with prednisolone at a dose of 6 mg per day. She had never been treated with any immunosuppressive agent other than prednisolone. She was a former smoker; 1 pack per day for 10 years until 35 years old (10 pack-years). She had no occupational dust inhalation such as silica or asbestos. At the age of 47, fine crackles were auscultated on bilateral lower lung fields of her back, and ILD was recognized by chest computed tomography (CT) (Fig. 1A). At 50 years old, a productive cough appeared and sputum cytology resulted in class V, suspicious of squamous cell carcinoma. The primary lesion was present in the ILD area of the right lower lobe (Fig. 1B). No evidence of distant metastasis was observed. She received right middle and lower lobectomy. The pathological diagnosis was well-differentiated squamous cell carcinoma, T2N1M0, stage IIB. Subsequently, she received two cycles of adjuvant chemotherapy with carboplatin and docetaxel. After a complete response for 1 year, the disease relapsed locally, and she finally died of cancer at 3 years after the surgery.

## Discussion

To assess clinical features of SqLC associated with SSc, we searched the literature on PubMed by crossing the keywords “lung cancer” and “systemic sclerosis”. The search period was from 1956 to 2014. We also evaluated some additional references from the retrieved articles. From more than 250 reports, we found a total of 39 cases of SqLC associated with SSc [2, 8-25]. Of these, 19 cases were not described in clinical detail or only limited description was available because of epidemiological studies focusing on all types of malignancies [21-24] or on mortality and causes of death [25]. Although several other epidemiological studies have also reported lung cancer associated with SSc, histology was not described [7, 26-30] or SqLC was not included [31-34].

The clinical characteristics of the 21 well-documented cases of SqLC associated with SSc, including the present case, are summarized in Table 1. Fifteen patients (71%) were less than 60 years old at the SqLC diagnosis, and the average age was 57 years. Unfortunately, detailed smoking history was available in only nine cases: three never smokers, two light smokers (less than 10 pack-years), and four heavy smokers (more than 30 pack-years). ILD was recognized in 11 (65%) of 17 described cases. In addition, of eight cases in which tumors developed in a peripheral lung field, seven cases (88%) had ILD. The interval between SSc and SqLC diagnosis is noteworthy, the onset of SSc preceded, and after the progress of many years, SqLC was diagnosed in most cases. The average duration of the interval between SSc and SqLC was 12 years. Three cases had simultaneous development of these conditions, and SSc appeared after the diagnosis of SqLC in only one case.

**Table 1:** Clinical Characteristics of Reported Squamous Cell Lung Cancers Associated With Systemic Sclerosis

Case (Ref)	Author	Year	Age	Sex	Smoking history	ILD	Location in lung field	Onset of SSc from the diagnosis of cancer (years)
1 [8]	Tomkin	1969	42	F	0.1 pack/day	No	Central	-4
2 [9]	Monti	1973	55	F	ND	Yes	Peripheral	-13
3 [9]	Monti	1973	74	F	ND	Yes	Central	-24
4 [10]	Talbott	1979	44	M	ND	Yes	Peripheral	-4
5 [11]	Roumm	1985	47	M	ND	ND	ND	-30
6 [11]	Roumm	1985	50	F	ND	ND	ND	-25
7 [11]	Roumm	1985	68	M	ND	ND	ND	-6
8 [12]	Focan	1985	70	M	ND	No	ND	0
9 [13]	M’Raihi	1988	59	F	ND	Yes	Peripheral	-11
10 [14]	Goodfield	1988	57	M	Heavy	No	Peripheral	4
11 [15]	Winkelmann	1988	46	M	Never	ND	Unclear	-30
12 [16]	Enzenauer	1989	59	F	30 years	Yes	Central	0
13 [17]	Yoshida	2001	60	F	ND	Yes	Peripheral	-11
14 [18]	Pontifex	2007	59	M	100 pack-year	Yes	ND	-1
15 [18]	Pontifex	2007	61	M	30 pack-year	Yes	ND	-25
16 [18]	Pontifex	2007	76	F	50 pack-year	No	ND	-29
17 [19]	Kundu	2012	55	M	Never	Yes	Peripheral	-4
18 [20]	Gangopadhyay	2013	54	M	ND	No	Unclear	0
19 [2]	Colaci	2013	40	M	Never	No	Central	-21
20 [2]	Colaci	2013	71	M	Yes	Yes	Peripheral	-7
21	Kanaji	2015	50	F	10 pack-year	Yes	Peripheral	-14

ILD: interstitial lung disease; SSc: systemic sclerosis; ND: not described.

In the present case, SqLC occurred in the area of ILD after a 14-year period from the diagnosis of SSc. From a collection of cases, SqLC associated with SSc has several clinical characteristics. First, the average age at the diagnosis of SqLC is 57 years, which is much younger than reported for patients without SSc in previous large studies (66 - 69 years old) [35, 36]. Second, although SqLC is generally associated with smoking history, it can occur in patients with SSc even if they are never or light smokers. Third, two-thirds of the available cases had ILD. In addition, most cases in which SqLC developed in a peripheral lung field had ILD. Fourth, SqLC generally does not occur until a long period after the diagnosis of SSc; the average of this interval reaches 12 years.

Although smoking is a known risk factor for ILD, lung involvement of SSc is also usually recognized as ILD [7]. Interestingly, SqLC occurred in the area of ILD in seven of nine described cases with ILD. These findings suggest that ILD might be related to the development of SqLC from underlying SSc. Consistent with this, an increased relative risk of cancer has been reported in the presence of ILD [11]. Several mechanisms have been proposed regarding lung cancer development from underlying ILD. First, it has been speculated that the terminal bronchiolar epithelium has proliferative potential and finally may lead to malignant transformation, particularly alveolar cell carcinoma [37]. Second, altered immunologic processes may be associated with the development of a malignancy superimposed on the ILD [37]. Third, DNA damage induced by reactive oxygen species (ROS) might initiate malignant transformation [1], although there is no report demonstrating direct evidence for this. Fourth, reduced clearance of carcinogens in the area of ILD may be causative or the atypical epithelium may be susceptible to carcinogens [38].

On the other hand, SqLC occurred in central lung fields in two never or light smokers without ILD [2, 8]. Some mechanisms other than ILD should therefore exist in a subset of SSc cases. In this regard, decreased or altered anti-neoplastic immune functions such as a decrease in the number of killer T cells may result in cancer development [39]. Many types of cells (fibroblasts, endothelial cells, lymphocytes, monocytes, mast cells and platelets), growth factors (transforming factor (TGF)-β, platelet-derived growth factor, etc.), and cytokines (tumor necrosis factor, interleukin-1, interleukin-2, etc.) are considered important to characterize SSc features such as the vascular intimal proliferation, microvascular obliteration, and ILD [40, 41]. Some growth factors such as TGF-β, cytokines, and chemotactic agents are also involved in cellular injury, repair, and genetic damage, and they are thought to contribute to development of lung cancer in addition to ILD [39, 42].

The theories mentioned above may explain why SSc is a cause of cancer development. On the other hand, some humoral factors released from cancer cells may lead to characteristics of SSc. In a case of simultaneous onset of adenosquamous cell carcinoma and SSc, TGF-β was strongly positive in tumor cells and adjacent macrophages [43]. Recently, the occurrence of SSc through the mechanism of paraneoplastic autoimmunity has also been proposed [44]. An association between the presence of anti-topoisomerase I and cancer in patients with SSc has been reported [45]. Furthermore, a close temporal relationship between onset of cancer and SSc has been reported in patients with anti-RNA polymerase I/III antibodies [46]. These findings suggest that SSc may appear as a paraneoplastic syndrome. In the present review, simultaneous onset of SqLC and SSc was observed in three cases [12, 16, 20]. In one case, SqLC occurred within 4 years after the onset of SSc [14]. These cases might be paraneoplastic. However, in the remaining 17 cases, SSc preceded and cancer appeared after an average of 12 years, suggesting that SSc cases suggestive of paraneoplastic syndrome are uncommon, unlike dermatomyositis and polymyositis [47, 48].

In conclusion, SqLC generally occurs in the area of ILD a long period after the diagnosis of SSc; the average duration of this interval reaches 12 years. Although SqLC is usually associated with smoking history, it may occur even in younger and never or light smokers in patients with SSc.
